# Internalized Stigma in Patients with Bipolar Disorder: A Cross-sectional Study on Its Associations with Sociodemographic, Marital and Clinical Characteristics

**DOI:** 10.1192/j.eurpsy.2024.208

**Published:** 2024-08-27

**Authors:** S. Tanyeri Kayahan, S. Vahip

**Affiliations:** ^1^Psychiatry, Yalvaç State Hospital, Turkish Republic Ministry of Health, Isparta; ^2^Psychiatry, Ege University School of Medicine, İzmir, Türkiye

## Abstract

**Introduction:**

Bipolar disorder (BD) is a chronic and complex affective disorder among top diseases that cause disability worldwide. Internalized stigmatization is a process including the awareness of negative stereotypes adopted by the society, participation in and internalization of these judgements, associated with impaired social functionality. Studies examining internalized stigma and related factors in BD is limited.

**Objectives:**

In this study, it is aimed to investigate the associations between internalized stigmatization and clinical characteristics, as well as sociodemographic and marital features of patients with BD.

**Methods:**

This observational and cross-sectional study was conducted at a specialized affective disorders clinic in a university hospital between November 2020 and March 2021. During routine follow-up, each consecutive patient with BD was invited and a total of 118 were included in the study. Information about sociodemographic, marital and clinical characteristics of patients was collected through a prepared data form and follow-up documents. Internalized Stigma of Mental Illness Scale (ISMIS) was administered to assess internalized stigma. Statistical analysis of data was conducted by SPSS version 25 and a statistical significance level of p<0.05 was determined.

**Results:**

Mean ISMIS total score of the sample was 56.50 ±13.65. Multiple linear regression was used to test the predictors of higher ISMIS scores. Being currently unemployed (p=0.012, Β=0.208), shorter BD duration (p<0.001, Β=0.302) and presence of inter-episode residual symptoms (p=0.004, Β=0.248) predicted higher ISMIS total. Younger age (p=0.002, Β=0.264), being female (p=0.007, Β=0.226) and absence of mania dominance (p=0.019, Β=0.190) predicted higher alienation scores. Presence of inter-episode residual symptoms predicted both stereotype endorsement (p<0.001, Β=0.320) and perceived discrimination (p<0.001, Β=0.358). Younger age (p=0.001, Β=0.281) and total number of depressive episodes (p=0.015, Β=0.212) also predicted perceived discrimination. Shorter BD duration and absence of seasonality predicted higher ISMIS social withdrawal, while history of hospitalization predicted higher ISMIS stigma resistance.

**Conclusions:**

Our study demonstrated similar mean ISMIS total scores to the findings previously reported in Türkiye, while roughly lower than results in the international literature. Considering that internalized stigmatization was increased in earlier stages of BD and in younger patients, as well as in patients with inter-episode residual symptoms, it might be important to implement psychosocial interventions for internalized stigmatization and appropriate psychoeducation programs in the earlier periods of BD. Therefore a multidimensional and holistic approach towards internalized stigmatization may positively contribute to the functionality of patients with BD.

**Disclosure of Interest:**

None Declared

